# STAT1 Is Required for Decreasing Accumulation of Granulocytic Cells via IL-17 during Initial Steps of Colitis-Associated Cancer

**DOI:** 10.3390/ijms22147695

**Published:** 2021-07-19

**Authors:** Yael Delgado-Ramirez, Itzel Baltazar-Perez, Yamileth Martinez, Blanca E. Callejas, Itzel Medina-Andrade, Jonadab E. Olguín, Norma L. Delgado-Buenrostro, Yolanda I. Chirino, Luis I. Terrazas, Sonia Leon-Cabrera

**Affiliations:** 1 Unidad de Biomedicina, Facultad de Estudios Superiores-Iztacala, Universidad Nacional Autónoma de México, Tlalnepantla, Edo. De México 54090, Mexico; sonr_sanjlv@hotmail.com (Y.D.-R.); itzel.bp.050997@gmail.com (I.B.-P.); stat1kowt@gmail.com (Y.M.); aletse_bianca@hotmail.com (B.E.C.); itzelmedina.andrade@gmail.com (I.M.-A.); je.olguin@iztacala.unam.mx (J.E.O.); nlbuenrostro@gmail.com (N.L.D.-B.); irasemachirino@gmail.com (Y.I.C.); literrazas@unam.mx (L.I.T.); 2 Laboratorio Nacional en Salud, Facultad de Estudios Superiores-Iztacala, Universidad Nacional Autónoma de México, Tlalnepantla, Edo. De México 54090, Mexico; 3 Carrera de Médico Cirujano, Facultad de Estudios Superiores Iztacala, Universidad Nacional Autónoma de México, Tlalnepantla, Edo. De México 54090, Mexico

**Keywords:** colitis-associated cancer, STAT1, IL-17, MDSCs

## Abstract

Signal transducer and activator of transcription 1 (STAT1) acts as a tumor suppressor molecule in colitis-associated colorectal cancer (CAC), particularly during the very early stages, modulating immune responses and controlling mechanisms such as apoptosis and cell proliferation. Previously, using an experimental model of CAC, we reported increased intestinal cell proliferation and faster tumor development, which were consistent with more signs of disease and damage, and reduced survival in STAT1^-/-^ mice, compared with WT counterparts. However, the mechanisms through which STAT1 might prevent colorectal cancer progression preceded by chronic inflammation are still unclear. Here, we demonstrate that increased tumorigenicity related to STAT1 deficiency could be suppressed by IL-17 neutralization. The blockade of IL-17 in STAT1^-/-^ mice reduced the accumulation of CD11b+Ly6C^low^Ly6G+ cells resembling granulocytic myeloid-derived suppressor cells (MDSCs) in both spleen and circulation. Additionally, IL-17 blockade reduced the recruitment of neutrophils into intestinal tissue, the expression and production of inflammatory cytokines, and the expression of intestinal STAT3. In addition, the anti-IL-17 treatment also reduced the expression of Arginase-1 and inducible nitric oxide synthase (iNOS) in the colon, both associated with the main suppressive activity of MDSCs. Thus, a lack of STAT1 signaling induces a significant change in the colonic microenvironment that supports inflammation and tumor formation. Anti-IL-17 treatment throughout the initial stages of CAC related to STAT1 deficiency abrogates the tumor formation possibly caused by myeloid cells.

## 1. Introduction

Signal transducer and activator of transcription 1 (STAT1) is part of the Janus kinase (JAK/STAT) signaling pathway and mediates important events associated with mucosal homeostasis, intestinal immune function, and cancer progression. Many lines of evidence suggest that STAT1 acts as a tumor suppressor molecule in various malignancies [1,2], prompting antiproliferative and proapoptotic responses and enhancing antitumor immunity [2].

Colitis-associated colorectal cancer (CAC) frequently develops in subjects with inflammatory bowel diseases (IBDs), such as ulcerative colitis (UC) and Crohn’s disease (CD) [3]. Colon cancer patients with high STAT1 expression have better clinical outcomes than those with low STAT1 expression in the colon tissue [4,5,6]. A reduction in STAT1 expression and/or a loss of its activation occurs in malignant colorectal cancer cells [7]. Additionally, it has been reported that STAT1 promotes apoptosis and suppresses the proliferation in colon tumor cells [8]. This is related to the ability of STAT1 to induce the expression of members of the cell surface death receptor family and their ligands [9,10] and a number of proapoptotic and cell-cycle genes, such as caspases, BCL-2, BCL-XL, p27, and p21WAF1 [11]. Previously, we demonstrated the role of STAT1 as a tumor suppressor molecule in inflammation-associated carcinogenesis [8]. During the very early stages of CAC initiation, STAT1 knockout mice (STAT1^-/-)^ presented elevated colonic epithelial cell proliferation, decreased apoptosis, and the overexpression of the antiapoptotic protein Bcl-2 in the colon [8]. In addition, an accumulation of granulocytic cells in the spleen and an increased production of IL-17A, IL-17F, and IL-22 cytokines in the colon, compared to WT mice were observed. These changes were associated with the higher susceptibility of STAT1^-/-^ animals to inflammation and tumor formation, compared to their WT counterparts [8]. The tumor suppression function of STAT1 has been associated with cell growth arrest, apoptosis, and inhibition of angiogenesis, but our studies suggest that STAT1 plays an important role in immune response modulation during inflammation-associated cancer. However, the mechanisms through which STAT1 regulates immune responses during the onset of CAC are not entirely understood.

IL-17 is a proinflammatory cytokine mainly produced by CD4+ T helper cells, and it has been related to colon cancer progression. IL-17 levels are increased in tumor tissues and in the sera of colon cancer patients [12,13], and a high expression of IL-17 in tumor regions has been proposed as an indicator of prognosis in patients with colorectal carcinoma [14]. IL-17 promotes angiogenesis and cell migration by stimulating the production of several chemokines and growth factors, such as vascular endothelial growth factor (VEGF), matrix metalloproteinase (MMP)-9, MMP-13, chemokine receptor 6 (CCR6), and prostaglandin E_2_ (PGE2), promoting tumorigenesis, angiogenesis, and metastasis [13]. Several studies indicate that IL-17 favors the accumulation of myeloid-derived suppressor cells (MDSCs) during cancer [15,16,17]. These cells are a heterogeneous population composed of immature myeloid cells, which are observed frequently in the circulation and are increased at tumor sites, where they suppress immune responses and promote tumor growth [18]. In mice, MDSCs are grouped into polymorphonuclear (PMN-MDSCs) and mononuclear (M-MDSCs) subsets based on the expressions of Ly6C and Ly6G markers [17,18]. Despite MDSCs being considered a consequence of tumor progression, in recent years, it has been demonstrated that the accumulation of granulocytic immature myeloid cells also occurs under inflammatory conditions and predisposes the individual to the development of cancer [19]. IL-17 induces the production of IL-6 by stromal and tumor cells, thus activating the STAT3 pathway, which favors the expression of inflammatory factors important for the regulation of leukocyte infiltration in inflammatory tissues [20]. On the contrary, STAT1 is recognized as an inhibitor of the Th17 differentiation pathway, and Th17 responses are affected by STAT1-activating cytokines such as IFN-γ and IL-27 [21]. Additionally, it has been well established that IL-17 promotes the activation and accumulation of neutrophils and PMN-MDSCs at sites of inflammation [22]. Published data show that chronic inflammation is present in almost all the observed sporadic colorectal adenoma samples and that IL-17A is upregulated since the adenoma stage and its level remains high until the cancer stage [23,24]. Therefore, IL-17 can promote the initial stages of tumorigenesis, creating a positive feedback loop leading to the accumulation of more neutrophils and PMN-MDSCs in the colon. However, the mechanism of the initial events enabling Th17 responses and PMN-MDSCs accumulation, ultimately leading to the formation of the feedback loop promoting CAC development, remains unclear.

Our previous studies have demonstrated that a lack of STAT1 renders mice highly susceptible to CAC, which correlates with rapid and extensive intestinal damage, increased cell proliferation in the early stages of injury-induced tumor formation, and reduced apoptosis in advanced tumors. To further characterize the contribution of STAT1 deficiency in CAC development, in this study, we examined alterations in myeloid cell recruitment along the adenoma–carcinoma sequence. Our study reveals that the effect of host STAT1 deficiency on tumor development can be reversed upon IL-17-blockade treatment, which correlates with a decreased accumulation of CD11b+ Ly6C^low^Ly6G+ cells and neutrophils and a remarkable reduction in tumor growth. These data show the tumor suppressor functions of STAT1 in colorectal cancer development and progression, which rely on the limitation of the IL-17-directed accumulation of granulocytic cells. Thus, STAT1 signaling may represent a potential target for therapeutic intervention during the initial stages of colorectal cancer.

## 2. Results

### 2.1. Deregulated Myeloid Cell Recruitment during Early Stages of Tumor Development in STAT1 Deficiency

**Figure 1:**
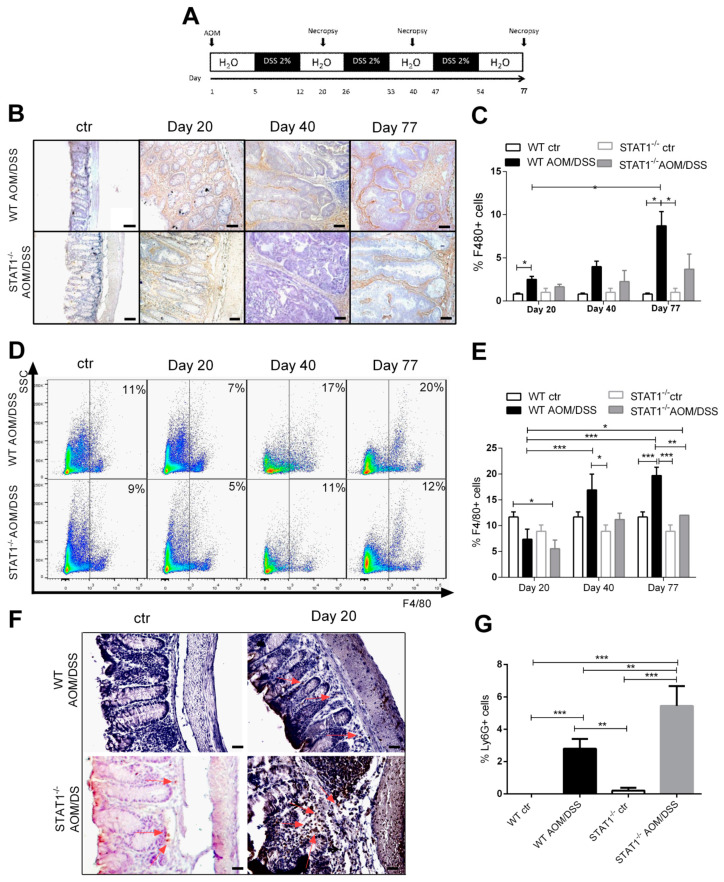
Macrophage populations are reduced in STAT1^-/-^ AOM/DSS-treated mice: (**A**) schematic time schedule of azoxymethane (AOM) and dextran sodium sulfate (DSS) administration. After the initial AOM injection (12.5 mg/kg), DSS was given in drinking water for 7 days followed by 14 days of regular drinking water. Mice were sacrificed on days 20, 40 (early tumor development), and 77 (late tumor development) post-AOM injection; (**B**) representative microscopic images showing immunohistochemical staining of F4/80 macrophages in colon tumor sections from WT and STAT1^-/-^ mice treated with AOM/DSS at different times of tumor progression Scale bars: 50 µm; (**C**) percentage of cells stained positive for F4/80; (**D**) representative dot plots showing CD11b+F4/80+ cell populations in spleens of WT and STAT1^-/-^ mice treated with AOM/DSS at the indicated time intervals; (**E**) percentages of CD11b+ F4/80+ cells in the splenocytes of WT and STAT1^-/-^ mice treated with AOM/DSS at different times of tumor progression; (**F**) immunohistochemical staining and (**G**) percentage of Ly6G+ cells in colon tissues in day 20. The quantification of Ly6G+ cells was performed using ImageJ software v.1.48 by counting cells in 10 high-powered fields from at least three slides per animal. Scale bars: 50 µm.The red arrowheads indicate Ly6G localization. Data shown are the mean ± SE of four or five individual mice per group per day of analysis and are representative of three individual experiments with similar results per day of the analysis. * *p* < 0.05, ** *p* < 0.01, *** *p* < 0.001.

Previously, we determined that STAT1 deficiency rapidly hastens the development of CAC through an azoxymethane (AOM)/DSS regiment, with greater histological damage and increased cell proliferation, as well as reduced apoptosis, during early CAC development, which affects the animal’s chance of survival [8]. One potential mechanism that contributes to accelerated tumorigenesis in STAT1^-/-^ mice is the increased recruitment of myeloid cells. Abnormalities in the myeloid cell compartment can promote tumor progression as associated with inflammation [18]. To test this hypothesis, we analyzed alterations in macrophage accumulation during the course of the disease for 20-, 40-, and 77-day periods in STAT1^-/-^ and WT mice, as an approximation of different stages of tumor progression (Figure 1A). The macrophage compartment identified by F4/80 expression in the colon tissue of WT mice was found to be expanded in colon cancer mice, compared with noncancer mice (Figure 1B,C). On the contrary, the expansion of macrophages was not increased in STAT1^-/-^ AOM/DSS animals, compared to STAT1^-/-^ naïve mice. However, the prevalence of F4/80+ cells was 2.4-fold higher in WT AOM/DSS mice, compared to STAT1^-/-^ AOM/DSS mice during advanced stages of tumor development (day 77) (Figure 1C). We also observed increases in the frequency of CD11b+F480+ cells in the spleens of tumor-bearing WT mice, compared with STAT1^-/-^ AOM/DSS animals in the early (day 20) and advanced stages of tumor development (day 77) (Figure 1D,E). In contrast, no differences in this cell population were observed in tumor-bearing STAT1^-/-^ mice. Thus, the absence of STAT1 results in impaired macrophage recruitment during colitis cancer progression.

To determine if STAT1 deficiency alters other subsets of myeloid cells, we examined the accumulation of neutrophils via Ly6G expression in the intestine during tumor development. Neutrophil frequency was increased in STAT1^-/-^ AOM/DSS animals at day 20 (early stage of tumor development), compared with WT AOM/DSS mice (*p* < 0.01, Figure 1F,G). Previously, we showed that STAT1^-/-^ AOM/DSS mice developed severe inflammation, with significant infiltration of leukocytes into the mucosa and extensive ulceration and erosion, particularly in the middle to the distal colon at day 20 after AOM administration, compared with WT AOM/DSS animals [8]. These data show enhanced neutrophil accumulation in the absence of STAT1 in the very early stages of tumor transformation. However, macrophage accumulation in the colon and spleen was reduced in STAT1^-/-^ AOM/DSS mice.

### 2.2. STAT1 Deficiency Increases the Accumulation of Myeloid-Derived Suppressor Cells (MDSCs) during Chronic Inflammation

**Figure 2:**
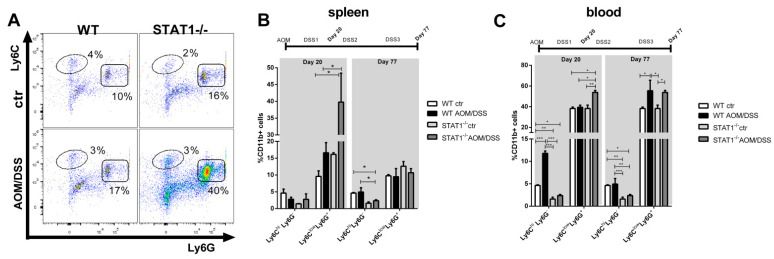
STAT1 deficiency increases the accumulation of CD11b+Ly6C^low^Ly6G+ granulocytic cells: (**A**) representative flow cytometry plots in the spleen are shown with Ly6C and Ly6G markers of gated living CD11b+ population cells on day 20. The bar graph illustrates the percentages of cell populations in the spleen (**B**) and blood (**C**) at the indicated time intervals (day 20 and day 77), gated as in Figure 2A. Data shown are the mean ± SEM of four or five individual mice per group per day of analysis and are representative of two individual experiments with similar results per day of the analysis. * *p* < 0.05, ** *p* < 0.01, *** *p* < 0.001.

MDSCs are characterized by potent immunosuppressive activity and the ability to promote tumor angiogenesis, tumor cell invasion, and metastases [18]. Additionally, MDSCs can contribute to tumor development associated with inflammation [19]. We asked what role, if any, these cells may have in tumor development associated with STAT1 deficiency. Our analysis revealed a significant increase in the accumulation of CD11b+ Ly6C^low^Ly6G+ cell populations in the spleen and blood of tumor-bearing STAT1^-/-^ mice, compared with WT mice at the very early stages of tumor transformation (Figure 2A–C). In the spleens of STAT1^-/-^ AOM/DSS animals, CD11b+ Ly6C^low^Ly6G+ cells were two times more prevalent, compared with WT AOM/DSS mice at day 20 (*p* < 0.05, Figure 2A,B). As CAC progressed, the accumulation of granulocytic cells decreased in the spleens of STAT1^-/-^ AOM/DSS animals, with no differences between tumor-bearing STAT1^-/-^ and WT mice at day 77 (Figure 2B). Similar trends were found in the blood of STAT1^-/-^ AOM/DSS animals at day 20, although to a lesser extent (Figure 2C). At day 77, increases in the CD11b+Ly6C^low^Ly6G+ cell populations were observed in the blood of both WT and STAT1^-/-^ AOM/DSS mice, with no differences between groups (Figure 2C).

The proportion of CD11b+Ly6C^hi^Ly6G- monocytic cells in the blood at day 20 was 2.5-fold higher in WT AOM/DSS mice, compared to STAT1^-/-^ AOM/DSS mice (*p* < 0.001, Figure 2C), and it decreased as CAC progressed. By contrast, the monocytic subset was significantly less abundant in STAT1^-/-^ mice throughout the experiment. These data suggest that the faster development of colon tumors in STAT1-deficient mice depends on the accumulation of granulocytic cells and neutrophil recruitment. Next, we investigated the mechanisms by which PMN-MDSCs accumulation leads to tumor promotion.

### 2.3. Early IL-17A Neutralization Inhibits Tumor Development in STAT1^-/-^ Mice

**Figure 3:**
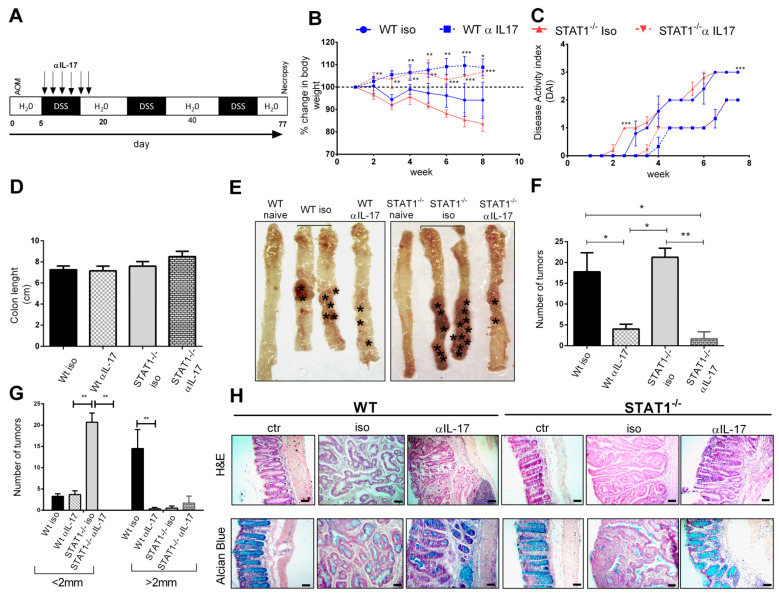
Therapeutic efficacy of IL-17 neutralization in STAT1^-/-^ mice during CAC: (**A**) WT or STAT1^−/−^ mice were injected with isotype control or anti-IL-17 neutralizing antibody from day 7 to 19 every other day (black arrows) after AOM/DSS administration. On day 77, the animals were euthanized to analyze tumor development; (**B**) weight changes in WT and STAT1^-/-^ mice with isotype and anti-IL17-treated mice. The mean weight of mice on day 0 of treatment was taken as 100% and was compared with the weekly weight until the end of the experiment; (**C**) the disease activity index (DAI) score was assessed on the indicated days for each animal and averaged per day for each group; (**D**) colon length, (**E**) macroscopic images of the colon (asterisks are pointing to the tumors), (**F**) number and (**G**) size of tumors 77 days after AOM injection; (**H**) colon sections stained with H&E as well as with Alcian blue to visualize goblet cells. Scale bars: 50 µm. Data shown are the mean ± SEM of at least four or five mice per group and are representative of two individual experiments with similar results per day of the analysis. * *p* < 0.05, ** *p* < 0.01, *** *p* < 0.001.

IL-17 plays an important role in the activation and recruitment of PMN-MDSCs and neutrophils in the tumor environment of many solid tumors [22]. In colorectal cancer, the production of IL-17 and TNF-α favors the recruitment of PMN-MDSCs and total MDSCs, promoting colorectal cancer (CRC) development and progression, and IL-17 leads to the accumulation of neutrophils in the colon [25]. Previously, we had determined that STAT1^-/-^ tumor-bearing mice produced higher levels of Th17 cytokines and expressed higher IL-17 transcripts in the intestine as CAC progressed than WT mice [8]. To evaluate if IL-17 was involved in this observed accumulation of CD11b+Ly6C^low^Ly6G+ cells and the increased rate of tumor development resulting from STAT1 deficiency, we examined the impact of anti-IL-17 treatment on MDSCs and neutrophil recruitment during CAC progression. Given that the CD11b+Ly6C^low^Ly6G+ cell populations were significantly increased in the spleens and blood of tumor-bearing STAT1^-/-^ mice at the very early stages of tumor transformation (Day 20, Figure 2), as well as the accumulation of neutrophils in the intestine (Figure 1F,G), we decided to perform an intraperitoneal injection of anti-IL-17 neutralizing antibody during the first DSS cycle (Figure 3A) and analyze the course of the disease for 77 days. First, we monitored weight loss, stool consistency, and bleeding via the disease activity index (DAI) score (Figure 3B,C). STAT1^-/-^ mice that received the IgG control antibody (isotype) displayed early enhancements in diarrhea and rectal bleeding, and a reduction in weight at the end of every DSS cycle, compared to similarly treated WT animals (Figure 3B,C). They also showed a significantly reduced survival rate during the first and third DSS cycles, as previously reported [8]. Additionally, upon necropsy on day 77, both STAT1^-/-^ and WT isotype-treated (iso) animals presented no differences in the number of tumors and the colon length between groups (Figure 3D–F). However, STAT1^-/-^ iso animals developed numerous small reddish polypoid tumors in the medial and distal zones of the colon, and more than the WT iso mice (Figure 3E,G). Interestingly, STAT1^-/-^ mice receiving anti-IL-17 antibody treatment developed significantly fewer tumors in the colon, compared with the STAT1^-/-^ mice that received isotype antibodies (1.6 vs. 21.25, *p* < 0.05), and survival was unaffected. In addition, the anti-IL-17 treatment also reduced the number and size of tumors in WT animals; however, the numbers of tumors smaller than 2 mm were similar between WT iso and WT anti-IL-17 groups. In addition, the colon tissues of WT iso and STAT1^-/-^ iso mice exhibited the classic chronic inflammation with glandular adenocarcinomas and fewer goblet cells, whereas the colon tissues of STAT1^-/-^ mice treated with anti-IL-17 antibody maintained normal histology as well as unchanged numbers of goblet cells (Figure 3H). These results demonstrate that the faster tumor development in STAT1^-/-^ mice could be stopped by IL-17 neutralization during the early stage of CAC.

### 2.4. IL-17 Neutralization Decreases the Recruitment of Neutrophils and CD11b+Ly6C^low^Ly6G+ Cell Populations

**Figure 4:**
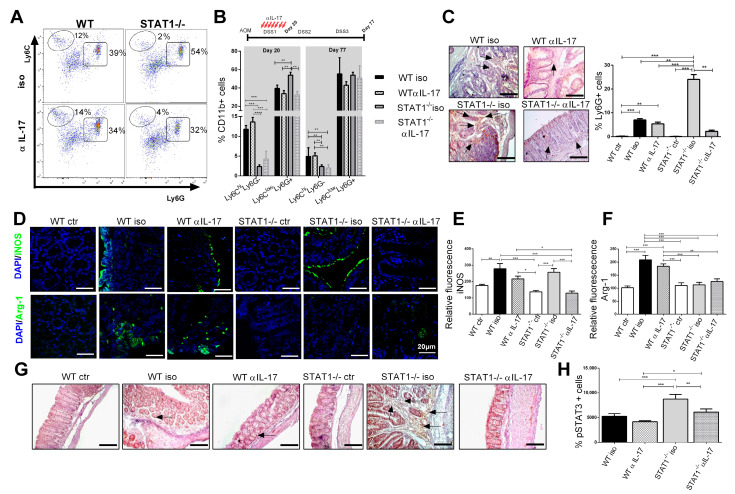
Anti-IL-17 treatment affects the recruitment of neutrophils and CD11b+Ly6C^low^Ly6G+ cell populations in STAT1^-/-^ mice. Mice were treated as in Figure 3A. Circulating cells were obtained from tumor-bearing WT or STAT1^-/-^ mice treated with anti-IL-17(αIL-17) or isotype (iso) antibodies. (**A**) representative flow cytometry plots are shown with Ly6C and Ly6G markers of gated living CD11b+ population cells in blood; (**B**) the bar graph illustrates the frequencies of CD11b+Ly6C^hi^Ly6G-and CD11b+Ly6C^low^Ly6G+ cell populations in blood at the indicated time intervals (day 20 and day 77), gated as in Figure 4A; (**C**) representative immunohistochemical staining of Ly6G in colon tissue at day 77. The arrowheads indicate Ly6G localization. Scale bars: 50 µm; (**D**) confocal representatives merged images of the immunofluorescence staining of the distal/affected colon tissue at day 77 using an anti-iNOS antibody (green) or anti-Arginase-1 antibody (green) and DAPI (blue) counterstaining. The images were captured in a confocal microscope at 63X. Scale bars = 20 μm. Relative fluorescence for iNOS (**E**) and Arginase-1 (**F**); (**G**) representative immunohistochemical staining of phosphorylated STAT3 in colon tissue at day 77. The arrowheads indicate pSTAT3 localization. Scale bars: 100 µm; (**H**) percentage of cells stained positive for pSTAT3. The quantification of Ly6G+ cells and pSTAT3+ cells was performed using ImageJ software v.1.48 by counting cells in 10 high-powered fields from at least three slides per animal. Data shown are the mean ± SEM of at least four or five mice per group and are representative of two individual experiments with similar results. * *p* < 0.05, ** *p* < 0.01, *** *p* < 0.001.

Next, we analyzed the impact of IL-17 neutralization on immune cell populations. In the early (day 20) and late (day 77) stages of tumor development, the accumulation of CD11b+Ly6C^hi^Ly6G- monocytic cells among CD11b+ cells were increased in WT mice treated or not with anti-IL-17 blocking antibody, compared with STAT1^-/-^ mice (*p* < 0.001, Figure 4A,B). On the contrary, the monocytic fraction was unaffected in iso and anti-IL-17 STAT1^-/-^ mice. The presence of CD11b+Ly6C^low^Ly6G+ granulocytic cells at day 20 was significantly reduced in STAT1^-/-^ mice following IL-17 blockade, compared with the STAT1^-/-^ mice that received the isotype antibody (*p* < 0.01, Figure 4A,B). However, no differences were observed at day 77 in this cell fraction between STAT1^-/-^ mice upon IL-17 neutralization (Figure 4B). Furthermore, we investigated whether blocking IL-17 affected neutrophil recruitment in the intestine. As expected, the infiltration of neutrophils in STAT1^-/-^ mice treated with anti-IL-17 blocking antibodies was reduced, compared with WT mice that received the same treatment (Figure 4C). One of the main characteristics of MDSCs in cancer is L-arginine depletion, which inhibits T-cell proliferation. Arginase-1 and iNOS activity promote tumor growth that escapes from immune surveillance through the depletion of L-arginine [26]; thus, we analyzed the levels of iNOS and Arginase-1 (Arg-1) expression in the intestine as a result of the activation of MDSCs in a pathological context. As shown in Figure 4D, WT iso mice presented an enhanced accumulation of Arg-1- and iNOS-positive cells in the colon, compared to naïve mice. However, Arg-1 expression was significantly reduced in the colon of STAT1^-/-^ iso animals, compared with WT (*p* < 0.01, Figure 1D,F). The administration of anti-IL-17 antibodies did not affect the expression of iNOS and Arg-1 in WT mice (Figure 4D–F). However, during the state of STAT1 deficiency, IL-17 neutralization resulted in a slightly reduced expression of iNOS, compared with STAT1^-/-^ mice that received isotype antibodies (Figure 4D,E).

The increased activity of STAT3 is common in colon cancer, and STAT3 phosphorylation has been related to tumor cell survival and proliferation, and tumor angiogenesis [27]. To analyze whether IL-17 may promote tumor growth via STAT3 during STAT1 deficiency, we evaluated STAT3 phosphorylation (pSTAT3) in colon tissue (Figure 4G). pSTAT3 was highly expressed in the colon of the STAT1^-/-^ iso group, compared to WT iso mice at day 20 (Figure 4G,H). The administration of the anti-IL-17 antibody significantly reduced pSTAT3 expression in STAT1^-/-^ animals, suggesting that IL-17 may promote tumor growth in the absence of STAT1 through the activation of the STAT3 signaling pathway.

### 2.5. Enhanced Inflammatory Microenvironment in STAT1^-/-^ Mice Is Controlled by IL-17 Blockade

**Figure 5:**
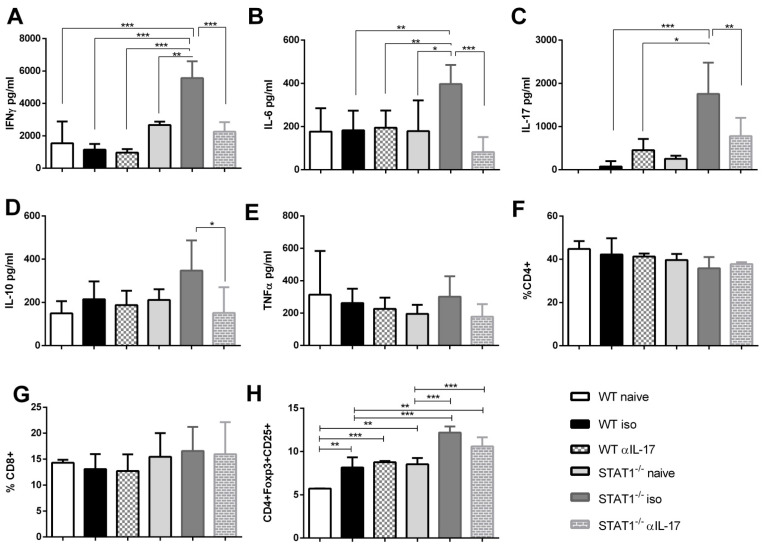
Treatment with anti-IL-17 antibody reduced the pro-inflammatory cytokine production in the spleens of STAT1^-/-^ mice. Mice were treated as in Figure 3A. In total, 1 × 10^5^ spleen cells were obtained at day 20 after AOM/DSS treatment and were stimulated with α-CD3 for 72 h to analyze the expressions of (**A**) IFN-γ, (**B**) IL-6, (**C**) IL-17A, (**D**) IL-10, and (**E**) TNF-α cytokines using the Mouse Th1/Th2/Th17 CBA Kit (BD^®^). Total spleen cells were analyzed by flow cytometry. Frequencies of total (**F**) CD4+ cells, (**G**) CD8+ cells, and (**H**) CD4+Foxp3+CD25+ T cells of tumor-bearing WT iso, STAT1^-/-^ iso or WT or STAT1^-/-^ mice treated with anti-IL-17 neutralizing antibody. Data shown are the mean ± SEM of three or four individual mice per group and are representative of two individual experiments with similar results. * *p* < 0.05, ** *p* < 0.01, ***p < 0.001.

STAT1 helps to maintain effective T-cell-mediated antitumor immune responses in different types of cancers [28,29]. The observed increase in granulocytic subsets during STAT1 deficiency at day 20 is probably the result of cytokines promoting their recruitment at a specific site. Therefore, we analyzed cytokine production by CD4+ T cells in the spleens of both isotype- and anti-IL-17-treated animals after a polyclonal stimulus (Figure 5A–E). We detected the early production of several inflammatory cytokines in the CD4+ T cells of tumor-bearing STAT1^-/-^ iso mice, with higher levels of IFN-γ, IL-6, and IL-17, compared with their WT counterparts (Figure 5A–C). Interestingly, the anti-IL-17 treatment significantly reduced the production of IFN-γ, IL-6, IL-10, and IL-17 in STAT1^-/-^α IL-17 animals, compared with STAT1^-/-^ iso. At this time, no differences in TNF-α production were observed. Moreover, no significant changes were observed in cytokine production in WTαIL-17 mice vs. WT iso mice (Figure 5A–E). Additionally, we analyzed the frequency of CD4+ and CD8+ T cells and Treg cells at days 20 and 77. Similar to CD4+ T cells, the frequency of CD8+ T cells was unaffected by IL-17 treatment in WT and STAT1^-/-^ mice (Figure 5F,G). Interestingly, the frequency of Treg cells, as assessed by Foxp3 and CD25 expression, was significantly increased in STAT1^-/-^ iso animals vs. WT on day 77 (Figure 5H). However, IL-17 neutralization did not affect the presence of Treg cells.

## 3. Discussion

STAT1 is a tumor suppressor molecule in CAC. In this study, we established that STAT1 deficiency deregulates the recruitment of myeloid cells via IL-17 during the early stages of tumor development. We determined that an IL-17 blockade prevents tumor advance almost completely during STAT1 deficiency. Mechanistically, the absence of STAT1 promotes an inflammatory microenvironment characterized by increased inflammatory cytokine production and recruitment of neutrophils, the accumulation of CD11b+Ly6C^low^Ly6G+ cells such as granulocytic MDSCs, and a greater expression of STAT3 in the intestine.

In the context of colon cancer, STAT1 is an antioncogenic molecule that acts in part through upregulating caspases [30], enhancing the expression of apoptosis-inducing ligand (TRAIL) [31] and cyclin-dependent kinase inhibitors [32], increasing phosphoinositide 3-kinase (PI3K) class IB signaling, downregulating the programmed cell death protein 4 (PDCD4) [33], enhancing the expression of the death receptor FAS and its ligand FASL [34], and downregulating the BCL2 family [2]. Even though STAT1 is a potential prognosis marker for colorectal cancer, the reports to date are variable. Recently, a correlation has been reported between shorter patient survival times and the high protein expression of STAT1 in early stage colorectal cancer [35]. Nonetheless, previous reports indicated a positive correlation of STAT1 expression and better prognosis [4,5]. The variable associations between survival and STAT1 expression may have arisen because the function of STAT1 has been analyzed in the early and late stages of cancer, without taking into consideration that the effects of this molecule may be stage-dependent. In our study, following CAC induction, STAT1-deficient mice developed accumulations of CD11b+Ly6C^low^Ly6G+ granulocytic cells in their spleens and mature neutrophils in the intestine, accompanied by the accelerated appearance of inflammation and tumor formation, advanced dysplasia, elevated colonic epithelial cell proliferation, and reduced apoptosis in the colon. This rapid onset of symptoms indicates that STAT1 slows down the inflammatory response in the early stages of the disease. For this reason, an understanding of the stage-dependent mechanisms of STAT1 in cancer is necessary for the use of this molecule in clinical diagnosis.

Several studies have linked the activation of STAT1 with MDSCs’ suppressive activity [36,37]. CD11b+Ly6C^low^Ly6G+ granulocytic cells were shown to be involved in angiogenesis and tumor growth in colorectal carcinoma, and MDSCs can infiltrate the tumor environment to activate Wnt/β-catenin signaling, thus promoting tumor cell proliferation [38]. The increased accumulation of CD11b+Ly6G+ granulocytic cells has been associated with augmented metastasis in breast cancer and head and neck squamous cell carcinoma during STAT1 deficiency [28,39]. It has been shown that the STAT1 protein is nitrated by nitric oxide (NO) derived from MDSCs during melanoma and pancreatic cancer, causing a disruption of the JAK/STAT1 signaling pathway. Consequently, dendritic cell (DC) antigen presentation was inhibited, suggesting that STAT1 nitration mediates MDSCs’ inhibitory effects on immune cells during cancer [40]. Our data showed reduced iNOS expression in the colon tissue after IL-17 neutralization in STAT1^-/-^ animals; thus, it is necessary to determine if antigen presentation was affected. Furthermore, the activation of STAT1 through IFN-γR could reduce PMN-MDSCs’ survival and suppressive activity by repressing the expression of the anti-apoptotic Bcl-2-related protein A1 [41]. Interestingly, the increased accumulation of MDSCs in the tissues of patients with inflammation or cancer was associated with an increased risk of tumor development [19]. In addition, during epidermal carcinogenesis, MDSCs accumulate in the skin, where they promote papilloma formation via IL-17-producing CD4+ cell recruitment as the initial step in the facilitation of tumorigenesis [19], suggesting that in inflammatory conditions, MDSCs can also act on immunosuppressive mechanisms. We have demonstrated that IL-17 neutralization during the early stages of tumor transformation results in the reduced recruitment of neutrophils, reduced CD11b+Ly6C^low^Ly6G+ cell populations, and lower iNOS expression in the intestine during STAT1 deficiency, which results in reduced tumorigenicity. Collectively, these data suggest the critical role of STAT1 in the early events of CAC, as it prevents the development of a favorable inflammatory microenvironment for tumor growth. However, it would be interesting to determine whether a reduction in MDSCs in STAT1^-/-^ mice would cause better antigen presentation and the priming of CD8+ T cells.

Different experimental studies have demonstrated that elevated IL-17 expression promotes colon cancer development [13,42,43]. In the Apc^Min/+^ mouse model, infection with enterotoxigenic Bacteroides fragilis (ETBF) triggers colitis and strongly induces colonic tumors. Antibody-mediated IL-17A neutralization blocked ETBF-induced colitis and tumorigenesis [44]. The inflammation and proliferation scores of IL-17A-deficient mice were significantly lower than those of WT mice in the AOM-DSS model of CAC [42]. IL-17 levels were increased in the sera and tumor tissues of colon cancer patients [23]. An in situ analysis of the density of IL-17+ cells in human colorectal cancer specimens showed that a high ‘‘Th17 signature’’ confers severely reduced disease-free survival after the resection of primary tumors [45]. Importantly, the therapeutic inhibition of Th17-associated cytokines may be of benefit in CAC. IL-17A neutralization restores responsiveness to chemotherapy in colon cancer and resensitizes cells to cytotoxic treatments [46]. Despite the evidence for the participation of IL-17 family members in colon cancer, the mechanisms that trigger IL-17 response at the onset of disease are unknown. This information is relevant for the use of IL-17 inhibitors as a therapeutic tool. Our data demonstrate a link between STAT1 deficiency and IL-17 in the development of CAC. Particularly, STAT1^-/-^ animals that received IL-17-blockade treatment during the first 20 days of CAC initiation were less susceptible to AOM/DSS-induced CAC. Considering that IL-17A is upregulated in the adenoma stage and its level remains high at the cancer stage; therefore, an anti-IL-17 treatment may be useful since the adenoma stage.

Suppressive M-MDSCs are characterized by the expression of iNOS and the production of nitric oxide (NO), while PMN-MDSCs produce reactive oxygen species (ROS) and arginase 1 (Arg1) as part of their immunosuppressive phenotype [47]. Both pathways promote the downregulation of T cell receptors, disrupting antigen presentation and anti-tumor T cell effector functions. In this study, we did not evaluate the immunosuppressive activity of CD11b+Ly6C^low^Ly6G+ cell populations but focused on the earlier effect of IL-17 neutralization in the accumulation of these and T cell populations under conditions of STAT1 deficiency. We found that although the granulocytic frequency was reduced after anti-IL-17 treatment in STAT1^-/-^ mice, the levels of CD4+ and CD8+ T cells and Treg cells were not affected. Notably, Arg-1 and iNOS expression in the intestine was significantly diminished in STAT1^-/-^ mice but not in WT mice during anti-IL-17 administration, which is consistent with the inhibition of tumor growth. It is possible that reductions in Arg-1 and iNOS expression may be associated with a reduced CD11b+Ly6C^low^Ly6G+ cell accumulation in STAT1^-/-^ mice. Accordingly, following exposure to a proliferative T cell environment, Ly6C+ monocytes, but not mature granulocytes, successfully express Arginase-1 [48]. Another means of inducing arginase production is the stimulation of macrophages with Th2 cytokines, such as interleukin-4 (IL-4), IL-10, and IL-13. To counteract the overproduction of toxic NO, the production of iNOS is often accompanied by arginase expression. Thus, the source of Arg-1 and iNOS in the intestine needs to be determined.

STAT1 is a critical regulator of Th1/Th17 differentiation. However, the relationship between STAT1 gain or loss of function, Th17 differentiation, and the increased susceptibility to CAC has not been investigated. Here, we demonstrated that the absence of STAT1 promotes IL-17 activity during the early stages of colon cancer via STAT3 and that IL-17 neutralization prevented tumor growth. This implies that STAT1 agonist could reduce IL-17 production or could block downstream of IL-17 and could be used as a therapy of CAC. However, this approach should be used in combination with chemotherapeutic drugs such as antiangiogenic agents, oxaliplatin, 5-fluorouracil, and anti-endothelial growth factor receptor antibodies. Further, a study on patients with inborn mutations in their STAT1 genes, and of the potential cross-regulation mechanisms between STAT1 and STAT3, may provide novel therapeutic strategies against colon cancer.

## 4. Materials and Methods

### 4.1. Mice

Eight- to ten-week-old female BALB/c mice were purchased from Harlan Laboratories (México) and maintained in a pathogen-free environment at the Facultad de Estudios Superiores Iztacala (FES-I), Universidad Nacional Autónoma de México (UNAM) animal facilities. STAT1^-/-^ female mice with a BALB/c genetic background were kindly donated by Dr. A.R. Satoskar. The animals were fed Purina Diet 5015 and water ad libitum. All experimental procedures were in strict accordance with the recommendations in the Guide for the Care and Use of Laboratory Animals of the National Institutes of Health (USA) and were approved by the Committee on the Ethics of Animal Experiments of the FES-I (UNAM): CE/FESI/102016/1096, 18-10-2016.

### 4.2. CAC Induction

Mice received an intraperitoneal (i.p.) injection of 12.5 mg/kg azoxymethane (AOM) (Sigma, St. Louis, MO, USA). Five days later, 2% dextran sulfate sodium (DSS, MW:40,000, Alfa Aesar, Tewksbury, MA, USA) in drinking water was administered ad libitum for 7 days. Mice were then provided regular water for 14 days and subjected to two more DSS cycles. To examine the early and late transformative steps in CAC, the mice were killed on days 20, 40 (early tumor development), and 77 (late tumor development) after AOM injection. Throughout the experiment, Disease Activity Index (DAI) scores were given to evaluate the clinical progression of CAC. DAI scores were calculated as the sum of changes in weight loss, compared to initial weight, stool consistency, and bleeding. During the necropsy, the colons were removed, weighed, and submitted for macroscopic inspection and histopathological examination.

### 4.3. IL-17 Neutralization In Vivo

BALB/c or STAT1^-/-^ mice were treated with 200 μg of In Vivo MAb anti-mouse IL-17A (clone: 17F3) (Bio X Cell, Lebanon, NH, USA), on days 7, 9, 11, 13, 15, 17, and 19 after AOM/DSS treatment and the control groups were treated with a similar concentration of isotype control via the intraperitoneal route.

### 4.4. Histological Analysis

For histological analysis, longitudinal sections from the large intestine were immediately fixed according to previously described protocols [8]. Colon sections with a thickness of 5 μm were stained with hematoxylin and eosin (H&E) to visualize morphology or with alcian blue to visualize goblet cells using an optical microscope (Axio Vert.A1, Carl Zeiss, Oberkochen, Germany). For immunohistochemical and immunofluorescence staining, sections were incubated overnight at 4 °C with primary antibodies against p-STAT3, Ly6G (both from GeneTex, Irvine, CA, USA), iNOS, Arginase-1 (both from Cell Signaling Technology, Danvers, MA, USA) and then developed following a conventional technique. Samples were analyzed with a ZeissVert.A1 conventional epifluorescence microscope and a LEICA TCS SP8X confocal microscope.

### 4.5. Flow Cytometry

Single-cell suspensions from spleens and the circulation obtained during the sacrifice were washed with PBS + 2% FBS and blocked with anti-CD16/CD32 antibodies (Biolegend, San Diego, CA, USA). Cells were stained with anti-F4/80, anti-CD11b, anti-Ly6C, and anti-Ly6G antibodies, or with anti-CD4, anti-CD8 and anti-CD25 antibodies (BioLeg-end, San Diego, CA, USA) for 30 min at 4 °C. Cells were washed twice and analyzed by flow cytometry using an Attune NxT (ThermoFisher^®^) cytometer. For intracellular staining, cells were washed and incubated in FOXP3 Fix/Perm Buffer Set (BioLegend, San Diego, CA, USA) for 30 min following the manufacturer’s instructions and then stained with anti-Foxp3 antibody (Becton Dickinson, San Jose, CA; USA). Then, 5000 gated events were captured and analyzed. Data were analyzed using the FlowJo software V X (Tree Star).

### 4.6. Quantification of Cytokines

Splenocytes from mice (1 × 10^5^ cells/mL) were plated in 96-well plates coated with anti-CD3 (Biolegend, San Diego, CA, USA) antibodies (2 µg/mL) in a complete RPMI medium in a humidified atmosphere containing 5% CO_2_ in air at 37 °C. After 72 h, the supernatants were harvested and stored at −70 °C until required. Cytokines were quantified using Mouse Th1/Th2/Th17 CBA Kit (BD^®^) (BD Biosciences, San Jose, CA, USA), following the instructions provided by the manufacturer.

### 4.7. Statistical Analysis

The data were analyzed with GraphPad Prism 5 by one-way ANOVA, followed by Tukey’s multiple comparisons test. All statistical tests were performed with 95% confidence intervals. The data are expressed as the means ± SEM, where * represents *p* < 0.05, ** represents *p* < 0.01 and *** represents *p* < 0.001.

## Data Availability

Not applicable.
